# Supplementary material for: Adverse Childhood Experiences Predict the Phenome of Affective Disorders and These Effects Are Mediated by Staging, Neuroimmunotoxic and Growth Factor Profiles
Source: Cells. 2022 May 7;11(9):1564. doi: 10.3390/cells11091564 (PMC9105661; doi:10.3390/cells11091564)
Supplement: Supplementary file 1 [file cells-11-01564-s001.zip › cells-1681305-supplementary.pdf]

ELECTRONIC SUPPLEMENTARY FILE (ESF)

Adverse childhood experiences are strongly associated with activation of M1 macrophage, T helper cell, neurotoxic and growth factor profiles and predict reoccurrence of illness and the phenome of affective disorders.

(1,2,3) Michael Maes, (1) Muanpetch Rachayon;; (1,4) Ketsupar Jirakran; (5,6) Pimpayao Sodsai; (5,6) Siriwan Klinchanhom; (7) Monojit Debnath; (8) Agnieska Basta-Kaim; (8) Marta Kubera; (1,9) Abbas F. Almulla; (1) Atapol Sughondhabirom.

(1) Department of Psychiatry, Faculty of Medicine, Chulalongkorn University and King Chulalongkorn Memorial Hospital, the Thai Red Cross Society, Bangkok, Thailand

(2) IMPACT Strategic Research Center, Barwon Health, Geelong, Australia

(3) Department of Psychiatry, Medical University of Plovdiv, Plovdiv, Bulgaria

(4) Maximizing Thai Children's Developmental Potential Research Unit, Department of Pediatrics, Faculty of Medicine, Chulalongkorn University, Bangkok, Thailand

(5) Center of Excellence in Immunology and Immune-Mediated Diseases, Department of Microbiology, Faculty of Medicine, Chulalongkorn University, Bangkok, Thailand

(6) Division of Immunology, Department of Microbiology, Faculty of Medicine, Chulalongkorn University, Bangkok, Thailand

(7) Department of Human Genetics, National Institute of Mental Health and Neurosciences, Bangalore, India

(8) Department of Experimental Neuroendocrinology, Polish Academy of Sciences, Kraków, Poland

(9) Medical Laboratory Technology Department, College of Medical Technology, The Islamic University, Najaf, Iraq.

ESF Table S1. The cytokines, chemokines and growth factors examined in the current study

Protein abbreviations	Gene Symbol	Protein name
**IL-1β**	IL1B	Interleukin-1β
**IL-1RA**	IL1RN	Interleukin-1 receptor antagonist
**IL-2**	IL2	Interleukin-2
**IL-4**	IL4	Interleukin-4
**IL-5**	IL5	Interleukin-5
**IL-6**	IL6	Interleukin-6
**IL-7**	IL7	Interleukin-7
**CXCL8**	CXCL8	C-X-C motif chemokine ligand 8 (IL-8)
**IL-9**	IL9	Interleukin-9
**IL-10**	IL10	Interleukin-10
**IL-12**	IL12	Interleukin-12
**IL-13**	IL13	Interleukin-13
**IL-15**	IL15	Interleukin-15
**IL-17**	IL17	Interleukin-17
**CCL11**	CCL11	Eotaxin
**FGF2**	FGF2	Fibroblast growth factor 2, Basic fibroblast growth factor
**G-CSF**	CSF3	Granulocyte Colony Stimulating Factor, Colony Stimulating Factor 3 (Granulocyte)
**GM-CSF**	CSF2	Granulocyte-macrophage colony-stimulating factor, Colony-stimulating factor 2
**IFN-γ**	IFNG	Interferon-γ
**CXCL10**	CXCL10	C-X-C motif chemokine ligand 8, Interferon gamma-induced protein 10 (IP10)
**CCL2**	CCL2	C-C Motif Chemokine Ligand 2 (MCP1)
**CCL3 / MIP-1α**	CCL3	Macrophage inflammatory protein-1 alpha, C-C Motif Chemokine Ligand 3
**PDGF**	PDGFA	Platelet Derived Growth Factor Subunit A
**CCL4 / MIP-1β**	CCL4	C-C Motif Chemokine Ligand 4, Macrophage Inflammatory Protein 1-Beta, Lymphocyte Activation Gene 1 Protein
**CCL5 /RANTES**	CCL5	C-C Motif Chemokine Ligand 5, Regulated Upon Activation, Normally T-Expressed, And Presumably Secreted
**TNF-α**	TNF	Tumor Necrosis Factor-Alpha
**VEGF**	VEGFA	Vascular Endothelial Growth Factor

Adapted from: Rachayon, M.; Jirakran, K; Sodsai, P; Klinchanhom, S.; Sughondhabirom, A.; Plaimas, K.; Suratanee, A.; Maes, M. Effects of cannabidiol on activated immune-inflammatory pathways in major depressive patients and healthy controls. Pharmaceuticals, 2022, in press.

ESF Table S2. The 9 immune and growth factor profiles employed in this study

Immune Profile	Members
**M1 macrophage**	IL-1β, sIL-1RA, IL-6, TNF-α, CXCL8, CCL3
**T helper-1**	IL-2, IFN-γ, IL-12
**T helper-2**	IL-4, IL-5, IL-9, IL-13
**T helper-17**	IL-6, IL-17
**IRS**	IL-1β, IL-6, TNF-α, CXCL8, CCL3, IL-2, IFN-γ, IL-12, IL-17, IL-15, G-CSF, GM-CSF, CXCL10, CCL5, CCL2
**CIRS**	IL-4, IL-10, sIL-1RA
**Neuroimmunotoxic**	IL-1β, IL-6, TNF-α, CXCL8, CCL3, IL-2, IFN-γ, IL-12, IL-17, CXCL10, CCL11, CCL5, CCL2
**Growth factors**	FGF, PDGF, VEGF
**T cell growth**	IL4, IL9, IL12, GM-CSF, IL-15

These profiles were computed as z unit based composite scores

IRS: immune-inflammatory response system; CIRS: compensatory immunoregulatory system

Adapted from: Rachayon, M.; Jirakran, K; Sodsai, P; Klinchanhom, S.; Sughondhabirom, A.; Plaimas, K.; Suratanee, A.; Maes, M. Effects of cannabidiol on activated immune-inflammatory pathways in major depressive patients and healthy controls. Pharmaceuticals, 2022, in press.

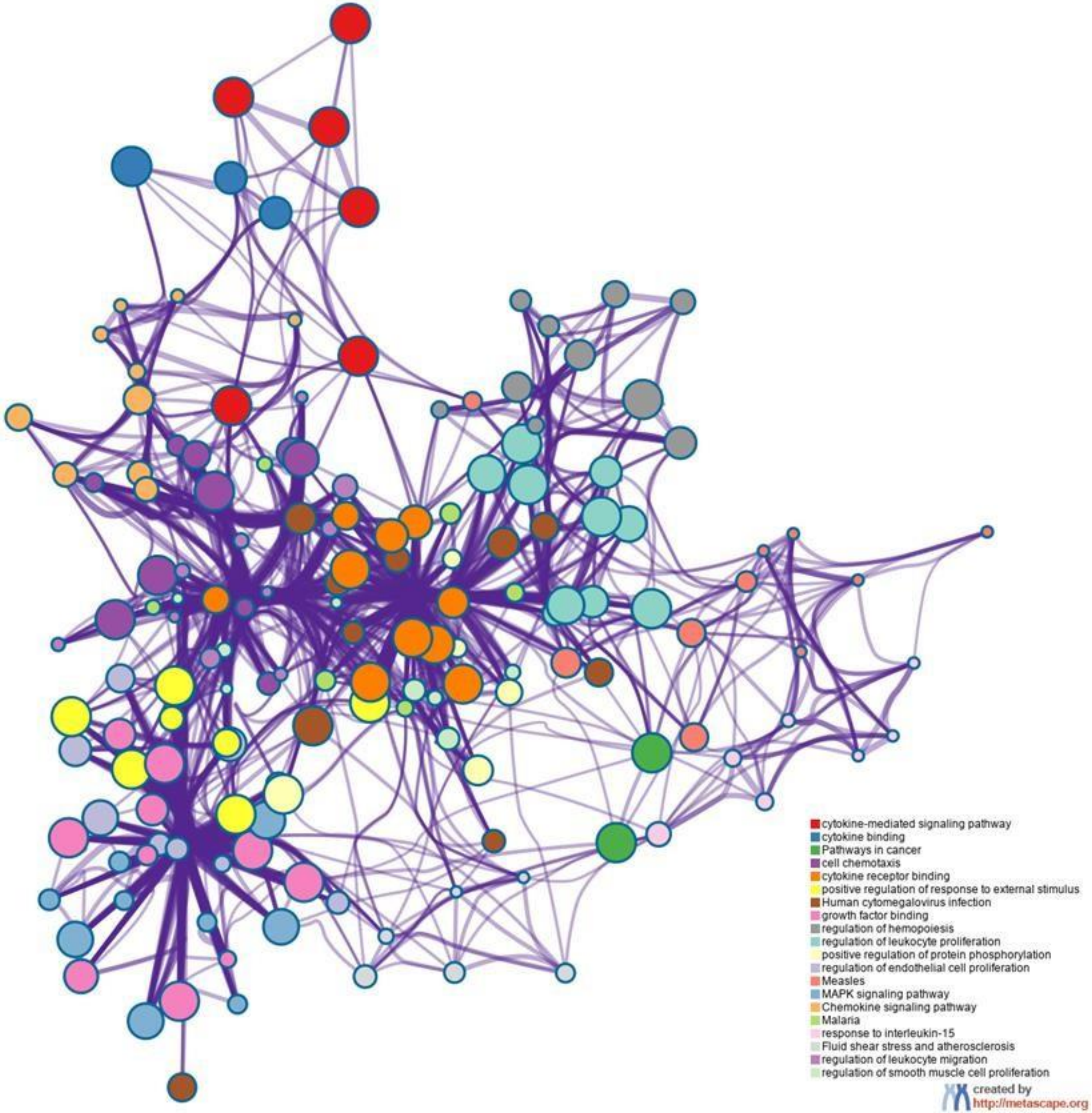
cytokine-mediated signaling pathway
cytokine binding
Pathways in cancer
cell chemotaxis
cytokine receptor binding
positive regulation of response to external stimulus
Human cytomegalovirus infection
growth factor binding
regulation of hemopoiesis
regulation of leukocyte proliferation
positive regulation of protein phosphorylation
regulation of endothelial cell proliferation
Measles
MAPK signaling pathway
Chemokine signaling pathway
Malaria
response to interleukin-15
Fluid shear stress and atherosclerosis
regulation of leukocyte migration
regulation of smooth muscle cell proliferation
created by
http://metascape.org

ESF Figure S1. Enriched ontology term clusters in the protein-protein-interaction network of Adverse Childhood Experiences. Circular nodes represent the terms that are overrepresented and their size reflects the amount of input genes. Colors identify clusters of terms and the colored labels describe the terms employed to characterize the clusters. The thickness of the bundled edges represents the similarity score with a threshold of 0,3. The network is generated using MetaScape.

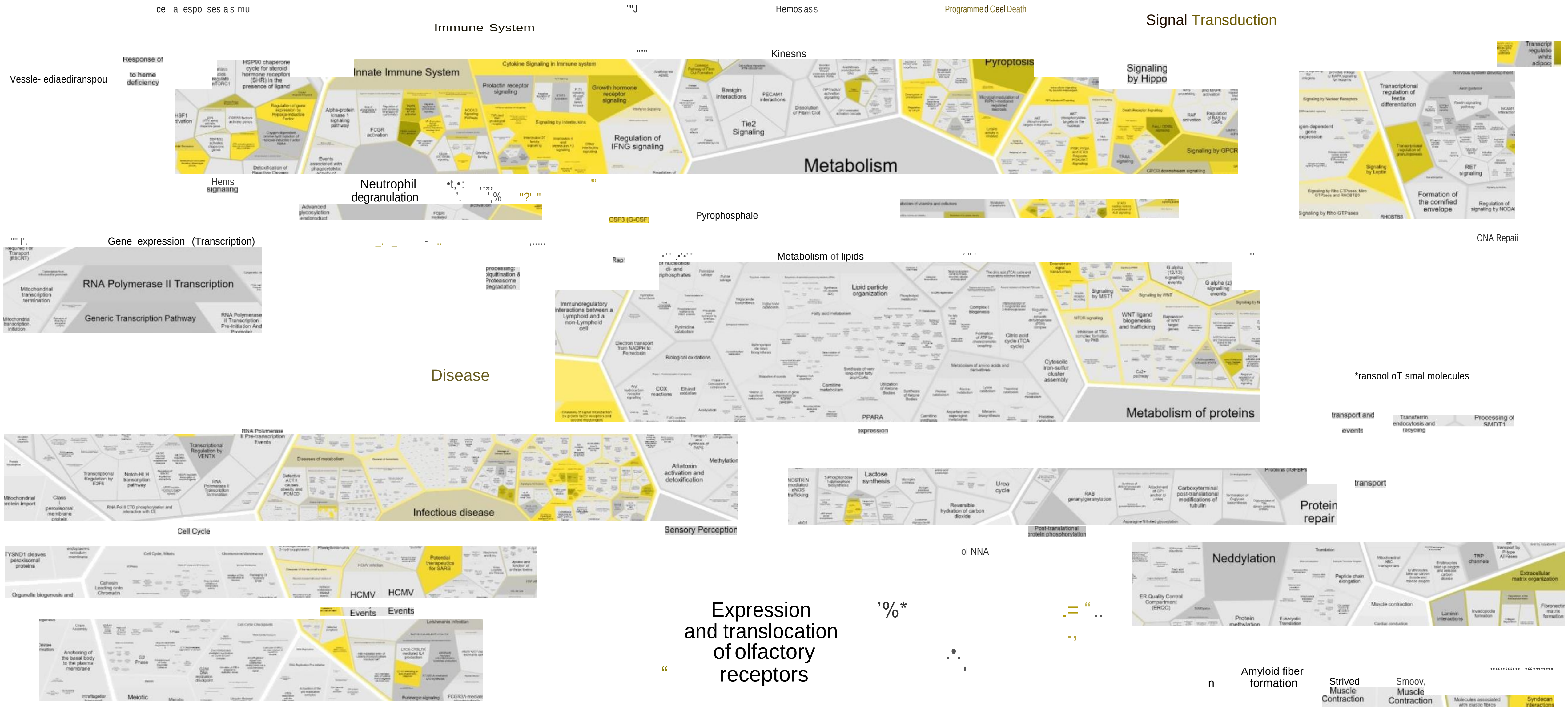

Immune System
Hemos ass
Programmed Ceel Death
Signal Transduction
Kinesns
Vessle- ediaediranspou
Response of to heme deficiency
HSP90 chaperone cycle for steroid hormone receptors (SHR) in the presence of ligand
Innate Immune System
Cytokine Signaling in Immune system
Prolactin receptor signaling
Growth hormone receptor signaling
Regulation of IFNG signaling
Basigin interactions
PECAM1 interactions
Tie2 Signaling
Pyroptosis
Signaling by Hippo
Metabolism
Hems signaling
Neutrophil degranulation
Pyrophosphale
CSF3 (G-CSF)
Gene expression (Transcription)
RNA Polymerase II Transcription
Generic Transcription Pathway
Metabolism of lipids
Lipid particle organization
Immunoregulatory interactions between a Lymphoid and a non-Lymphoid cell
Biological oxidations
Disease
Metabolism of proteins
ONA Repaii
*ransool oT smal molecules
transport and events
Infectious disease
Aflatoxin activation and detoxification
Cell Cycle
Sensory Perception
Lactose synthesis
Protein repair
ol NNA
Neddylation
HCMV Events
HCMV Events
Potential therapeutics for SARS
Expression and translocation of olfactory receptors
Amyloid fiber formation
Strived Muscle Contraction
Smoov, Muscle Contraction

ESF Figure S2. Voronoi visualization of the hierachical pathways overrepresented in the protein-protein interaction network of the genes that are upregulated in subjects with Adverse Childhood Experiences. This figure was constructed employing the Reactome pathway analysis.

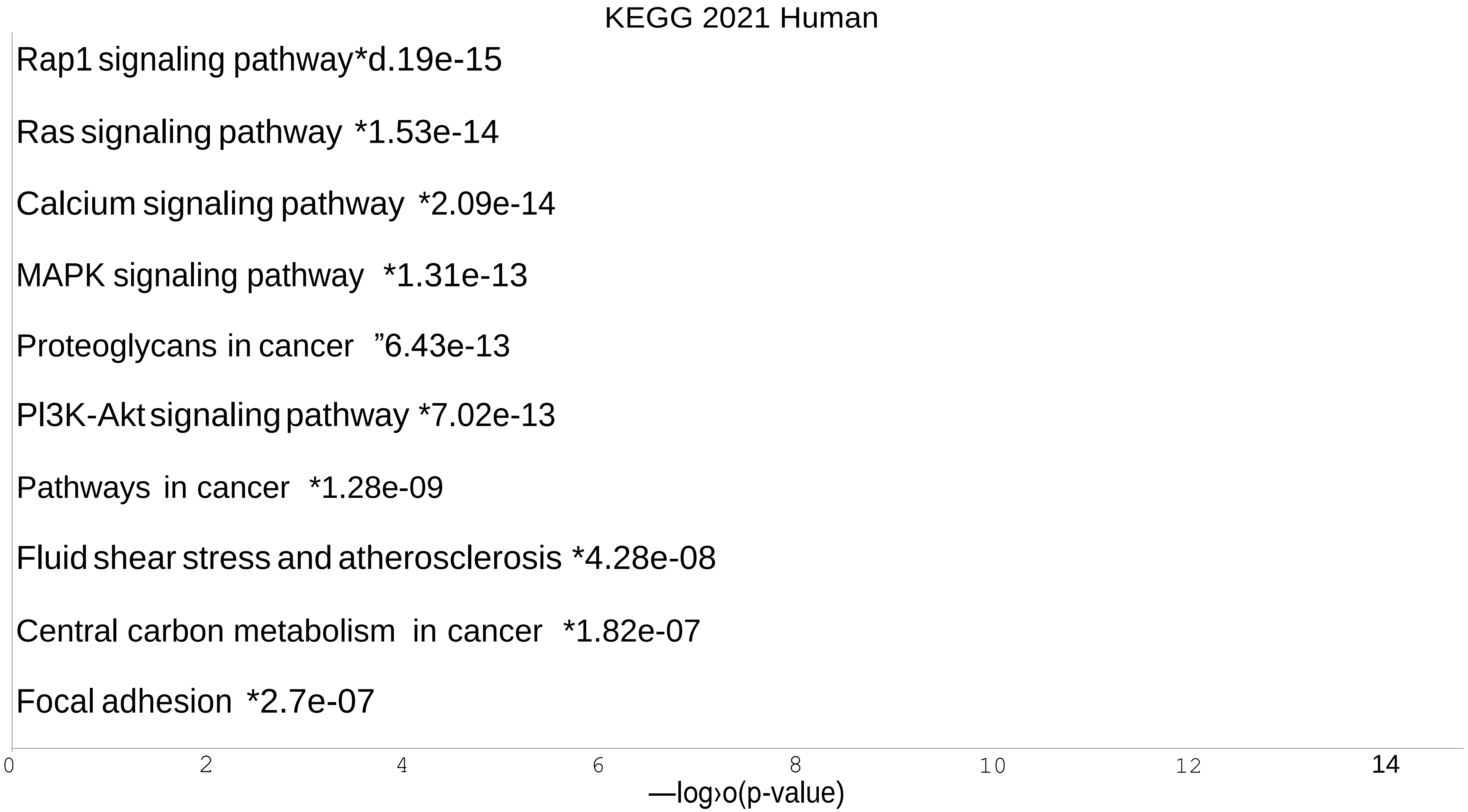
KEGG 2021 Human
Rap1 signaling pathway*d.19e-15
Ras signaling pathway *1.53e-14
Calcium signaling pathway *2.09e-14
MAPK signaling pathway *1.31e-13
Proteoglycans in cancer ”6.43e-13
PI3K-Akt signaling pathway *7.02e-13
Pathways in cancer *1.28e-09
Fluid shear stress and atherosclerosis *4.28e-08
Central carbon metabolism in cancer *1.82e-07
Focal adhesion *2.7e-07
0
2
4
6
8
10
12
14
—log›o(p-value)

ESF Figure S3. Bar graph made using Enrichr and visualized with Appyter showing the top 10 Kegg pathways which are overrepresented in the protein-protein network of the growth factor genes of Adverse Childhood Experiences.

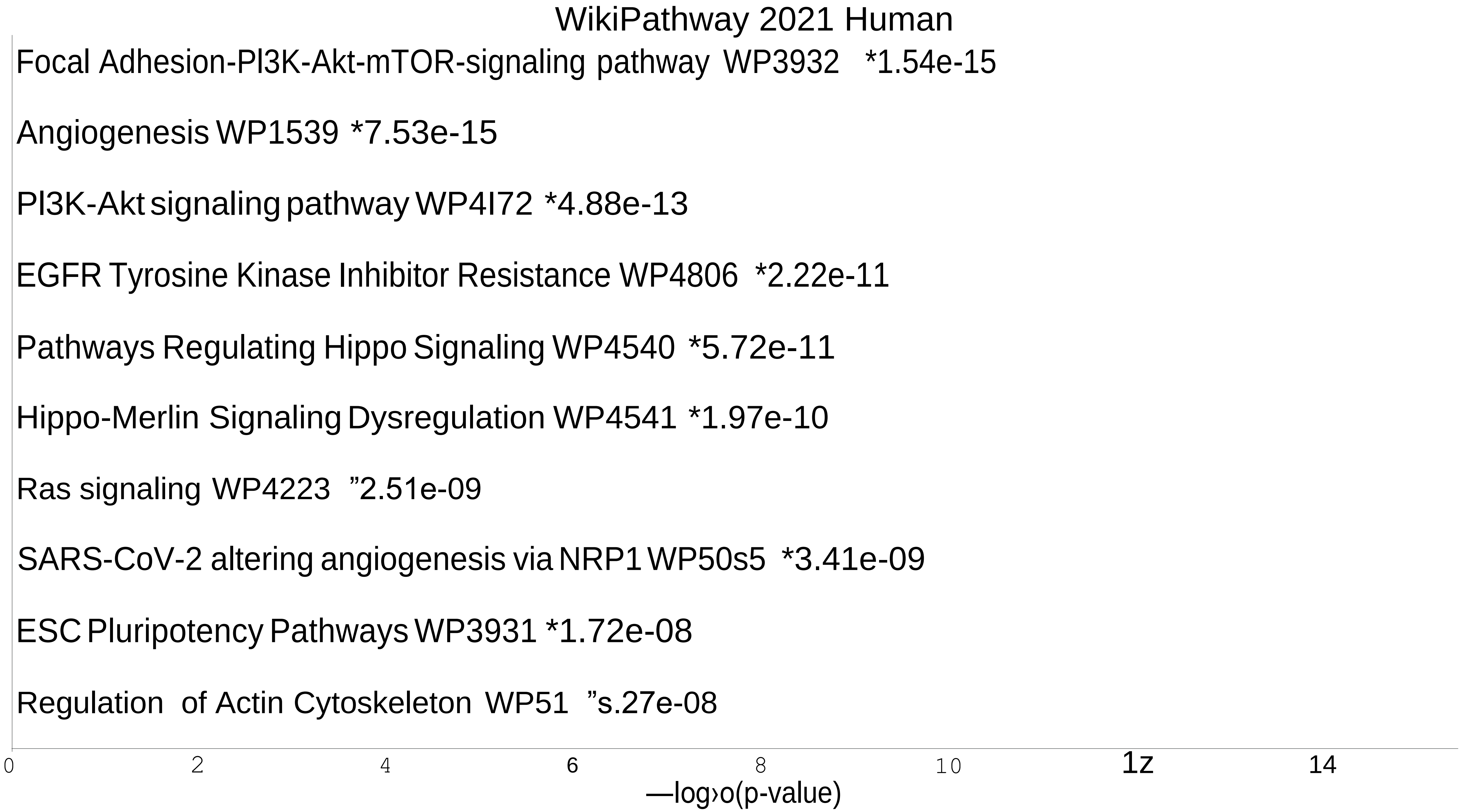
WikiPathway 2021 Human
Focal Adhesion-PI3K-Akt-mTOR-signaling pathway WP3932 *1.54e-15
Angiogenesis WP1539 *7.53e-15
PI3K-Akt signaling pathway WP4I72 *4.88e-13
EGFR Tyrosine Kinase Inhibitor Resistance WP4806 *2.22e-11
Pathways Regulating Hippo Signaling WP4540 *5.72e-11
Hippo-Merlin Signaling Dysregulation WP4541 *1.97e-10
Ras signaling WP4223 ”2.51e-09
SARS-CoV-2 altering angiogenesis via NRP1 WP50s5 *3.41e-09
ESC Pluripotency Pathways WP3931 *1.72e-08
Regulation of Actin Cytoskeleton WP51 ”s.27e-08
0
2
4
6
8
10
1z
14
—log›o(p-value)

ESF Figure S4. Bar graph made using Enrichr and Appyter showing the top 10 Wikipathways which are overrepresented in the growth factor network of Adverse Childhood Experiences.
